# Asynchronous online lecture may not be an effective method in teaching cardiovascular physiology during the COVID-19 pandemic

**DOI:** 10.1186/s12909-022-03217-w

**Published:** 2022-03-09

**Authors:** Weerapat Kositanurit, Sarocha Vivatvakin, Kasiphak Kaikaew, Pachara Varachotisate, Chuti Burana, Maneerat Chayanupatkul, Sekh Thanprasertsuk, Danai Wangsaturaka, Onanong Kulaputana

**Affiliations:** 1 Department of Physiology, Faculty of Medicine, Chulalongkorn University, 1873 Rama IV road, Pathumwan, Bangkok, 10330 Thailand; 2 Department of Pharmacology, Faculty of Medicine, Chulalongkorn University, 1873 Rama IV road, Pathumwan, Bangkok, 10330 Thailand; 3 Division of Academic Affairs, Faculty of Medicine, Chulalongkorn University, 1873 Rama IV road, Pathumwan, Bangkok, 10330 Thailand

**Keywords:** Asynchronous online lecture, Traditional lecture, Academic performance, Attitude, Undergraduate medical education

## Abstract

**Background:**

Asynchronous online lecture has become a common teaching method in medical education, especially during the COVID-19 pandemic. However, the effectiveness and students’ attitudes towards this method under this special circumstance have not been exclusively studied. Hence, we aimed to evaluate these aspects of cardiovascular physiology teaching in an undergraduate medical curriculum.

**Methods:**

We analysed and compared the academic achievement and attitudes of 613 medical students on cardiovascular physiology between pre-COVID-19 and COVID-19 years in which different teaching methods were implemented. In addition, we also explored the importance of teaching methods and teachers by subgroup analysis to evaluate whether they influenced the academic achievement and attitudes of students.

**Results:**

Overall students’ academic achievement was significantly higher when lectures were taught by the traditional method than by the asynchronous online method. Moreover, subgroup analysis revealed that teachers were also a factor influencing students’ academic achievement. Although most students had positive attitudes towards asynchronous online lectures, overall satisfaction was slightly higher when all lectures were taught by the traditional method than by the asynchronous online method.

**Conclusions:**

Asynchronous online lectures might not be an effective teaching method especially during the abrupt change in education. Under the ‘new normal’ medical education, not only teaching methods but also teachers are the essential keys to the success in academic achievement and attitudes of undergraduate medical students.

## Introduction

Lecture in a classroom has been a traditional and widely accepted method for large group teaching in medical education [1]. In the last two decades, the internet and its relevant technology were introduced and have become a part of our daily life, including its assistance in remote learning, i.e., electronic learning (e-learning). Also, e-learning has been applied in undergraduate and postgraduate medical education for more than a decade [2–4]. Several e-learning modalities have been developed by integrating emerging technologies, such as simulation, synchronous online learning, and asynchronous online learning [2]. The effectiveness of internet-based learning has been studied and revealed that internet-based learning is as effective as traditional lecture [5–7].

In 2020, the coronavirus disease 2019 (COVID-19) was an emerging infectious disease and became a worldwide pandemic within months. Affected countries implemented various policies aiming to control the outbreak, such as physical distancing, lockdown, and the closure of crowded places including schools and universities [8]. Therefore, the COVID-19 outbreak substantially accelerates the development of online teaching and learning to lessen the disruption of all levels of medical education [9]. Online teaching has become a preferable method, or sometimes the only feasible teaching method, for pre-clerkship education [10, 11].

COVID-19 outbreak emerged in Thailand in February 2020. The Ministry of Public Health of Thailand issued the policies for urgent prevention of disease transmission. Our institution, obliged by the government policies, also announced the regulations and guidelines for all academic activities during the pandemic. All teachings, with the exception of crucial clinical skills, must shift to an online method to keep physical distancing. In this situation, asynchronous online lecture might be one of the favourable teaching methods suitable for the ‘new normal’ standard during the outbreak [11]. We believed that asynchronous online lecture could mimic the traditional lecture since it fulfilled the fundamental basis of giving essential knowledge by teachers. Previous studies have shown that online teaching, when used, is as effective as on-site teaching, as seen by the improvement or similarity of students’ academic performance [6, 12–15]. Moreover, asynchronous online teaching is more favourable than on-site teaching since teachers (or instructors) and students can keep physical distancing and students can study without any time or space limit [11]. Nevertheless, there are some barriers interfering with the success of online teaching and learning, for instance, poor technological skills of teachers and inadequate institutional support, lack of time to develop e-learning materials, and feeling of disengagement between teachers and students [16].

Until now, the effectiveness and students’ attitudes towards asynchronous online teaching, particularly when online teaching is the only feasible teaching method for a large group of medical students during the pandemic, have not been studied exclusively. Thus, we aimed to investigate the effectiveness and students’ attitudes towards asynchronous online lectures compared with the traditional lectures on the topics of cardiovascular physiology in a pre-clerkship course of the undergraduate medical curriculum. In addition, we also aimed to compare students’ academic achievement and students’ attitudes between the two different teaching methods taught by the same teachers and among different teachers teaching similar topics.

## Materials and Methods

### Study population and study design

This study was designed to evaluate academic achievement and attitudes of the second-year medical students towards teaching methods used in the lecture topics on cardiovascular physiology, retrospectively for the pre-COVID-19 year and prospectively for the COVID-19 year. All students enrolled in the Cardiovascular System I course of the Faculty of Medicine, Chulalongkorn University, Bangkok, Thailand were included in the study unless they opted out. The Cardiovascular System I is the course taught in the second year of the medical curriculum comprising 16 lecture topics (24.5 h) and 17 non-lecture topics (44 h) involving anatomy, histology, biochemistry, physiology, and basic haematology. Of all lecture topics, 10 topics are relevant to cardiovascular physiology, listed in Table 1. Details of the course were described previously [17]. In this study, we aimed to analyse only topics related to cardiovascular physiology since they were taught solely by lectures, which were the focus of this study, and accounted for a majority of knowledge content covered by the course.

**Table 1:** The list of cardiovascular physiology topics and lecturers in the Cardiovascular System I course during pre-COVID-19 and COVID-19 years

Topics	Duration (hours)	Lecturers	
		**Pre-COVID-19**	**COVID-19**
1. Physical and electrical properties of the heart	2	A	F
2. Cardiac cycle & regulation of the heart	2		
3. Basic electrocardiogram	1	B	F
4. Circulatory dynamics	2	C	C
5. Circulatory control	2		
6. Regional circulation	1.5		
7. Physical activity & fitness	1	C	C
8. Exercise physiology	1		
9. Microcirculation	1	D	C
10. Pathophysiology of shock & heart failure	2.5	E	E

Letters A–F indicate each teacher (lecturer) teaching the topic in the indicated academic year

Concerning the topics of cardiovascular physiology presented in Table 1, some topics were taught by the same teachers while some topics were taught by different teachers between these two academic years. Nevertheless, all teachers were content experts in the respective cardiovascular topics. The topics covered in this course and the teaching duration of each topic were similar for both years. In the pre-COVID-19 year, all lecture topics were taught in a large lecture theatre by traditional lectures. Due to the COVID-19 pandemic and the implemented policies of physical distancing, all topics were shifted to asynchronous online lectures in the COVID-19 year. Asynchronous online lectures were newly recorded by the lecturers who taught those topics. Most lecturers used computer software, such as Microsoft PowerPoint and OBS Studio, to record lecture slides together with their narrations. One recorded lecture covered one topic and typically lasted 45–60 min. The online lectures, a.k.a. e-lectures, were uploaded to the available online platforms of the faculty (the MDCU E-learning website, available at http://e-learning.md.chula.ac.th) and of the university (the learning management system myCourseVille, available at https://www.mycourseville.com/). After the lecture sessions, an interactive question-and-answer (Q&A) session was conducted in the lecture theatre in the pre-COVID-19 year and via an online platform in the COVID-19 year to answer students’ questions and to interactively discuss any unclear points raised by the students. Of note, the Q&A session was a non-compulsory session.

### Measurement of academic achievement

Students’ academic achievement was assessed by the summative exam taking place approximately 6 weeks after the course completion. In both academic years, the summative exams of these courses were held on-site in the examination hall. With the epidemic prevention measures in the COVID-19 year, the exam was arranged similarly to the pre-COVID-19 year. The summative exam consisted of 5-option single best answer (SBA) questions, i.e., 112 questions for the pre-COVID-19 year and 108 questions for the COVID-19 year. Specifically, 64 out of 112 questions and 62 out of 108 questions directly aimed to assess the knowledge on cardiovascular physiology taught by lectures. The students’ summative scores were analysed and reported as a percentage of the total scores. Of note, the remaining questions were excluded as they were not relevant to our study objectives, i.e., they were used to assess knowledge on anatomy, histology, biochemistry, or cardiovascular physiology that taught by other teaching methods such as online live conference, online small group discussion, and asynchronous laboratory demonstration.

To ensure the equivalence of the summative assessment used in these two years, five medical doctors were asked to evaluate the relevance and difficulty of all examination items using the modified Ebel method, resulting in the minimum passing level of each item [18]. The items from the two academic years were pooled and randomly ordered so that the raters would not know to which year each item belonged. All raters independently determined the level of relevance (important/essential, acceptable, or questionable/additional) and difficulty (easy, moderate, or difficult) of each item. The expected percentage of correct answers was categorised into the 3 × 3 relevance-by-difficulty matrix table [18–20]. The percent-correct values and discrimination indices of all items were also analysed [21]. Items with negative discrimination indices, i.e., one item from each year, were excluded. The reliability coefficients of the tests were calculated using the Kuder-Richardson 20 (KR-20) formula [22]. The KR-20 value between 0.70 and 0.90 was accepted as a reliable assessment [23].

### Subgroup analysis of academic achievement

To identify if a difference in teachers/lecturers influences the academic achievement, we performed subgroup analysis by categorising each lecture topic into the topic taught by the same teachers (topics 4–8 and 10 in Table 1) or the topic taught by different teachers (topics 1–3 and 9 in Table 1). Then, a difference in academic achievement between academic years was analysed using unpaired *t* test.

### Measurement of student satisfaction

To assess student attitudes, course evaluation was performed at the end of the course through an online questionnaire, in which all items had been created, reviewed, and approved by the course committee, using Google Forms (the free web-based Google Docs Editors). Students could anonymously and voluntarily fill in the questionnaire. Students evaluated the quality of each lecture with a 5-point rating scale as follows: 5, excellent; 4, good; 3, average; 2, fair; and 1, poor. In the COVID-19 year, 8 statements in Table 2 were included to evaluate student agreement (strongly agree, agree, uncertain, disagree, or strongly disagree) on asynchronous online lectures and the Q&A session. Lastly, an open-ended question was included to specifically survey students’ attitudes towards online teaching. For the open-ended question, students’ comments were categorised into positive and negative comments by the consensus of 2 independent investigators.

**Table 2:** Students’ attitudes towards the asynchronous online lectures and the Q&A session

Statements	No. of responses	
	**Agree**	**Disagree**
1. Asynchronous online lecture is not different from the traditional lecture in helping you understand the lecture contents	137 (68.5%)	34 (17.0%)
2. Asynchronous online lecture helps you understand the lecture contents more efficiently than the traditional lecture	119 (60.1%)	24 (12.1%)
3. Asynchronous online lecture without face-to-face interaction does not cause any problems for understanding the lecture content	139 (69.8%)	29 (14.6%)
4. Asynchronous online lecture is more convenient than the traditional lecture	152 (76.0%)	24 (12.0%)
5. Asynchronous online lecture facilitates the management of your own schedule better than the traditional lecture	112 (56.3%)	54 (27.1%)
6. The Q&A session promotes your understanding of cardiovascular physiology contents	122 (63.2%)	12 (6.2%)
7. The Q&A session after a series of asynchronous online lectures with voluntary participation is satisfactory	140 (72.9%)	6 (3.1%)
8. For the next year, teaching by asynchronous online lecture is preferred over the traditional lecture	99 (50.5%)	57 (29.1%)

Data are presented as the number (percentage) of responses for each statement. Agree indicates ‘strongly agree’ and ‘agree’ while disagree indicates ‘strongly disagree’ and ‘disagree’. Of note, the agreement ‘uncertain’ is not presented in the table

The questionnaire had been carefully reviewed and approved by the Cardiovascular System I course committee and used to evaluate student satisfaction for several years, except for the 8 newly added statements in Table 2. In addition, all items in the questionnaire were assessed for content validity and reliability [24]. Content validity was determined using the index of item-objective congruence by 3 independent experts. Reliability was determined by Cronbach’s alpha. Our analyses showed that the indices of item-objective congruence of the 8 newly added statements and the satisfaction questions were 0.79 and 1.00, respectively. Cronbach’s alpha was 0.87 for the 8 newly statements and 0.75 for satisfaction questions representing an acceptable quality of the questionnaire.

### Subgroup analysis of student satisfaction

To identify if a difference in lecturers influences the student satisfaction, we also performed subgroup analysis into topics taught by same or different lecturers (Table 1). Then, a difference in student satisfaction between academic years was analysed using unpaired *t* test.

### Statistical analysis

Categorical data, i.e., sex of the participants, were demonstrated as frequency and percentage and analysed by Chi-square test. Unless otherwise indicated, continuous data were presented as mean, standard deviation (SD), and 95% confident interval (95% CI). To compare the difference between groups, continuous data including satisfactory level and the subgroup analyses were analysed by unpaired *t* test. Statistical analysis was performed by Microsoft Excel (version 16.0.13530.20368) and GraphPad Prism (version 9.0.0). A *p* value of < 0.05 was used to infer statistical significance.

## Results

**Fig. 1:**
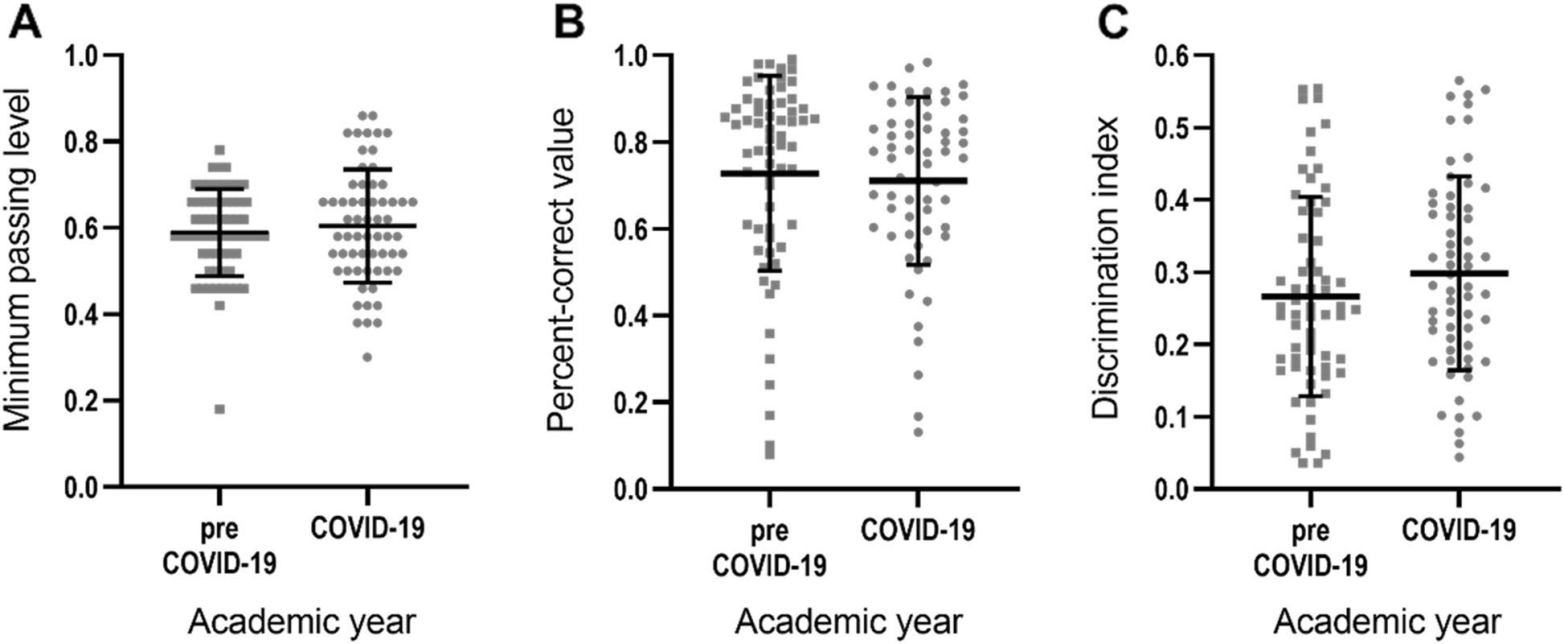
Item analysis of the summative exams. Lecture-pertinent items related to cardiovascular physiology topics in the summative exam were assessed for **A** minimum passing level (by modified Ebel method), **B** percent-correct value, and **C** discrimination index. Values of each item are shown by scattered dot plots, with lines indicating mean and error bars indicating SD. Differences between academic years were analysed by unpaired *t* test: **A**, *p* = 0.48; **B**, *p* = 0.65; and **C**, *p* = 0.19

Of 613 students enrolled in the study, 301 were those in the pre-COVID-19 year and 312 were those in the COVID-19 year. Baseline characteristics of the students did not differ between these two years, as shown in Table 3. As our main objective was to investigate the effectiveness of teaching methods between academic years by evaluating students’ academic achievement, we compared the three parameters (minimum passing levels, percent-correct values, and discrimination indices) between 2 academic years and found that they were not significantly different (Fig. 1). The reliability coefficients were also within the accepted range (KR-20 values = 0.83 for the pre-COVID-19 year and 0.84 for the COVID-19 year).

**Table 3:** Baseline characteristics of the students enrolled in the study

Student characteristics	Pre-COVID-19 year (*n* = 301)	COVID-19 year (*n* = 312)	*p* value
Sex (% female)	135 (45%)	160 (51%)	0.11
Age (years)	19.55 ± 0.77	19.60 ± 1.13	0.51
Credits registered before enrolling in the study	43.43 ± 1.36	43.49 ± 2.10	0.70
Credits achieved before enrolling in the study	43.00 ± 0.00	43.00 ± 0.00	1.00
Credits registered in the semester with Cardiovascular System I	21.00 ± 0.00	21.00 ± 0.00	1.00

Categorical data are presented as the number (percentage). Continuous data are presented as mean ± SD

### Academic achievement

**Fig. 2:**
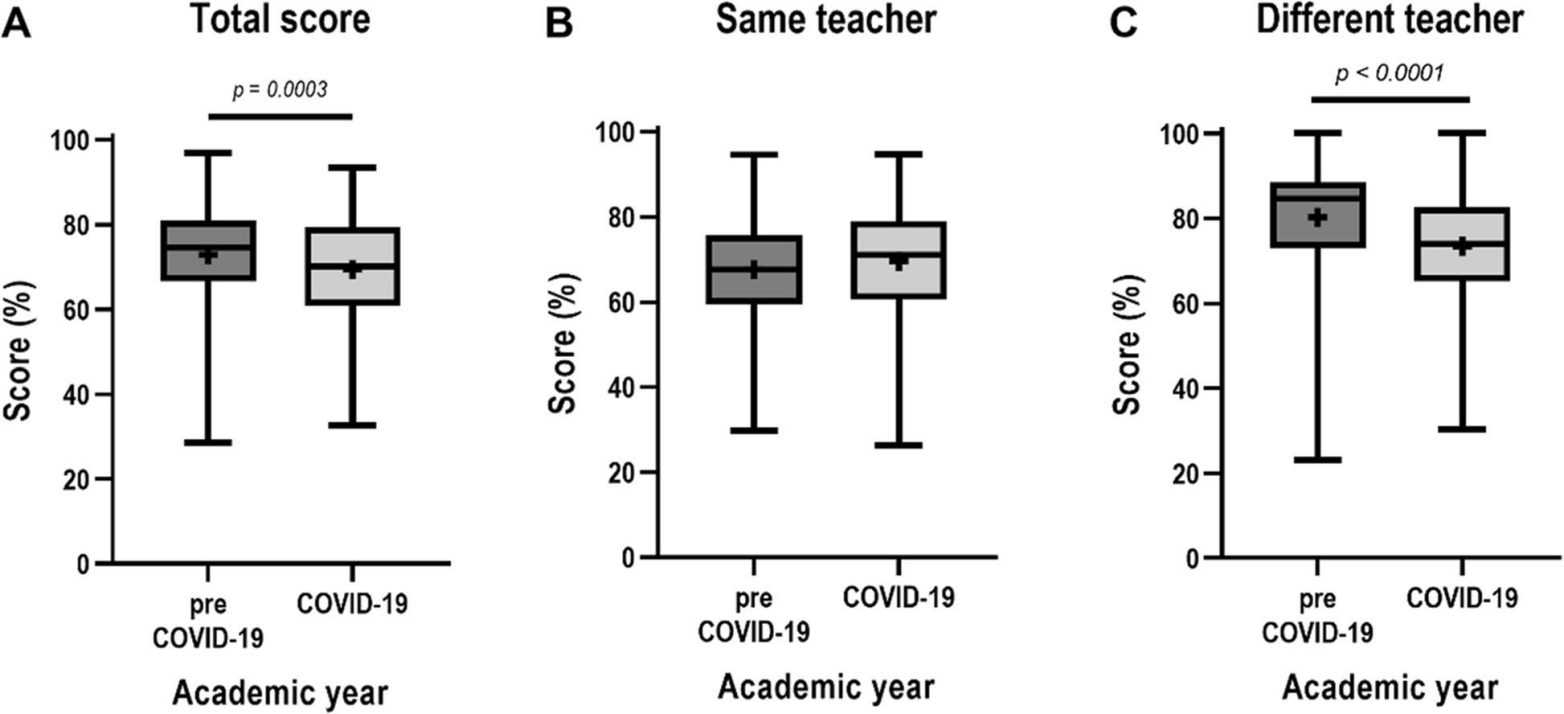
Students’ academic achievement. Students’ achieved scores in the summative exams are presented as follows: **A** total score, **B** score grouped by topics taught by the same teachers (topics 4–8 and 10 in Table 1), and **C** score grouped by topics taught by different teachers (topics 1–3 and 9 in Table 1). Data are presented by box-and-whisker plots using the Tukey method [boxes indicating an interquartile range (IQR), whiskers indicating 1.5 times IQR, lines in the box indicating the median, and plus signs in the box indicating mean]. Differences in scores between academic years in **A** and **B** were analysed by unpaired *t* test

Academic achievement of students, as indicated by the summative scores, was significantly higher in the pre-COVID-19 year (when lectures were taught by only the traditional on-site method) than in the COVID-19 year (when lectures were taught by the asynchronous online method) (Fig. 2A). The difference between means was 3.48 ± 0.97% (95% CI: 1.58 – 5.38). Subgroup analysis was performed to verify possible factors explaining the findings. The academic achievement of students in the lecture topics taught by the same teachers but different teaching methods was not significantly different between two academic years (Fig. 2B). The difference between means was -1.93 ± 1.00% (95% CI: -3.90 – 0.04). Interestingly, the subgroup analysis in topics taught by different teachers between two academic years showed that the score in the pre-COVID-19 year was significantly higher than that of the COVID-19 year (Fig. 2C). The difference between means was 6.79 ± 1.13% (95% CI: 4.58 – 9.00). Of note, this subgroup analysis (Fig. 2C) was a similar pattern observed in overall performance (Fig. 2A).

### Student satisfaction

**Fig. 3:**
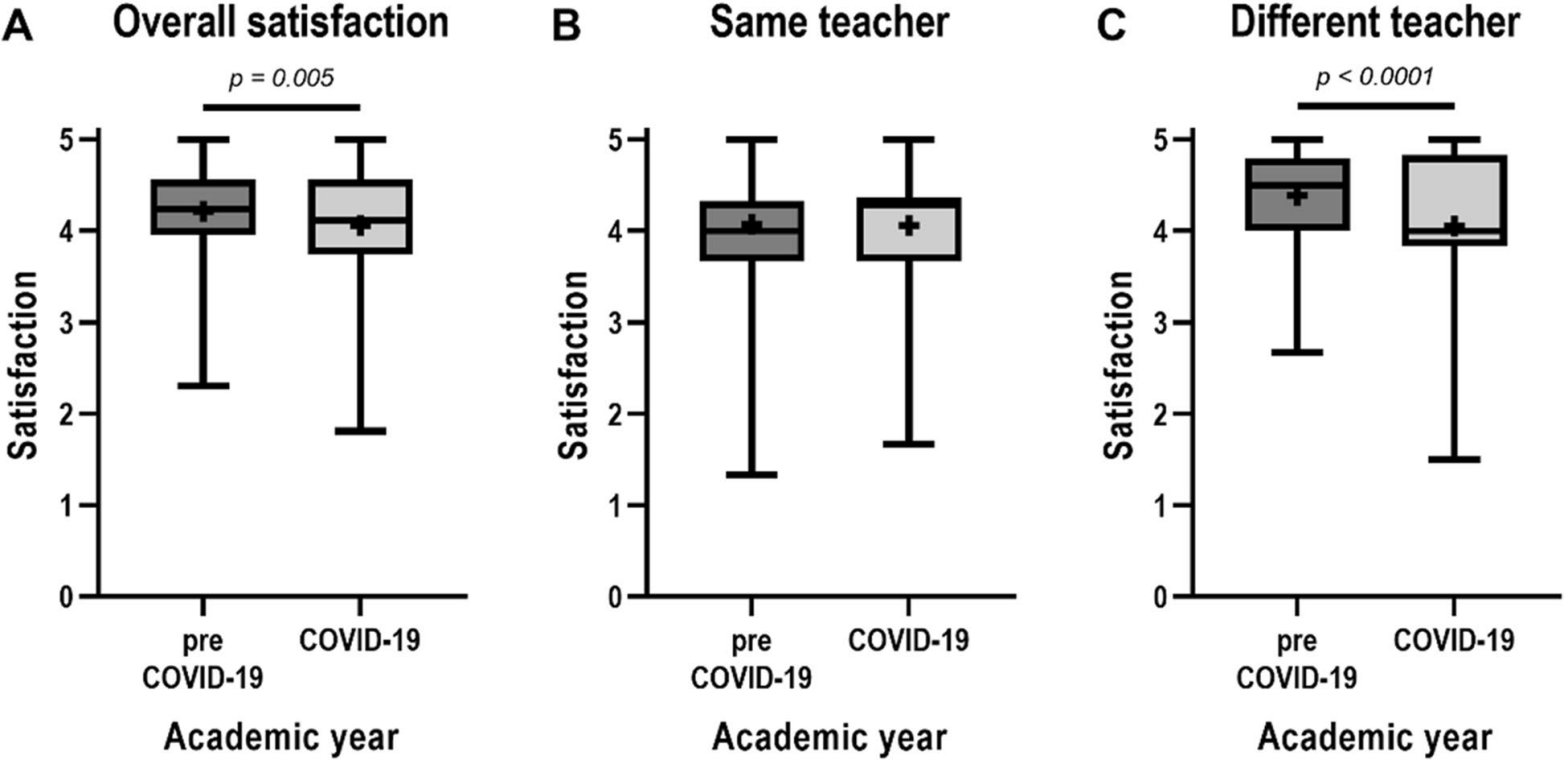
Students’ satisfaction with lectures. Students’ satisfaction evaluated by a 5-point rating scale is presented as follows: **A** overall satisfaction, **B** satisfaction grouped by topics taught by the same teachers (topics 4–8 and 10 in Table 1), and **C** satisfaction grouped by topics taught by different teachers (topics 1–3 and 9 in Table 1). Data are presented by box-and-whisker plots using the Tukey method [boxes indicating an interquartile range (IQR), whiskers indicating 1.5 times IQR, lines in the box indicating the median, and plus sign in the box indicating mean]. Differences in satisfactory levels between academic years in **A** and **B** were analysed by unpaired *t* test

Course evaluation via an anonymous questionnaire was received from 268 students (89.0%) in the pre-COVID-19 year and 204 students (65.4%) in the COVID-19 year. Overall satisfaction of lectures was significantly higher in the pre-COVID-19 year than in the COVID-19 year (Fig. 3A). In the subgroup analysis to compare different teaching methods by the same teachers, we found that student satisfaction was not different between these two years (Fig. 3B). On the other hand, the subgroup analysis to compare different teachers lecturing the same topics showed that student satisfaction was significantly higher in the pre-COVID-19 year than in the COVID-19 year (Fig. 3C). Of note, this pattern was in line with the students’ academic achievement presented in Fig. 2C.

We studied students’ perspectives towards asynchronous online lectures and found that the majority of students had positive attitudes towards asynchronous online lectures in all investigated aspects (Table 2). In general, the most satisfactory statements indicated a preference for the voluntary Q&A session and the convenience of asynchronous online lectures. Interestingly, the least agreement was the item concerning whether the asynchronous online lecture is more preferable than the traditional lecture, i.e., only a half of students rated in favour of the online lecture (Table 2). Lastly, students’ feedback towards asynchronous online lectures was presented in Table 4. Of note, some students reported drawbacks of asynchronous online lectures, such as the instability of the e-lecture platform and the longer time needed to finish each online lecture.

**Table 4:** Students’ comments on asynchronous online lectures

Positive feedback	Negative feedback
- The asynchronous online lecture can be learnt in various paces suitable for individual learning styles. (3) - Students can manage their own time effectively. (1) - Asynchronous online lecture is more convenient and flexible than the traditional lecture. (1)	- There are some technical issues, such as the instability of the e-learning platform and errors in internet connection. (24) - Students need more time to learn from asynchronous online lectures. (6) - Students lack the motivation to learn from asynchronous online lectures. (1)

Numbers in parentheses indicate the total number of responses

## Discussion

For our cardiovascular physiology lectures, the asynchronous online lecture was a teaching option before the COVID-19 outbreak but became the only teaching method during the outbreak due to an abrupt change in pandemic prevention policy. It was beyond our expectations that the academic achievement was higher in the pre-COVID-19 year (when all lectures were taught by the traditional on-site method) than in the COVID-19 year (when lectures were taught by the asynchronous online method). Our finding indicated that asynchronous online lecture may not be an effective substitute for the traditional on-site lecture. Possible explanation might be that a major limitation of asynchronous online lectures could be a lack of student–teacher interaction which might result in a loss of motivation and subsequently a decline in academic performance, in accordance with a previous study [25]. Therefore, an increase in interaction between students and teachers via interactive synchronous online sessions could be a solution. For example, three-fourths of the students in our study preferred the Q&A session which was synchronous and interactive. In addition, our notion was supported by previous studies demonstrating that highly interactive sessions could enhance students’ academic performance and that medical students preferred more interactive sessions for online learning such as case-based discussion and interactive live lectures [26–29]. Other explanations include an urgent shift to online learning without adequate preparation, limited resources, and insufficient technological support [30] which affect the quality of asynchronous online lecture.

Our finding also suggests that the teacher/lecturer might be another factor influencing student performance other than the teaching method or teaching platform alone. Our results revealed that different teachers/lecturers could bring about differences in academic outcomes of students. We believe that teaching skills might potentially be a key explanation. In higher education, good teaching skill has been the first rank characteristic of a good teacher [31], leading to the better academic achievement of medical students [32]. One way to improve teaching skills of the academic faculties is a continuum of faculty development. Obviously, a previous systematic review has shown that faculty development has a strong impact on enhancing faculties’ performance resulting in better students’ academic achievement [33].

A large body of research has shown that teaching methods have an influence on students’ attitudes towards engagement in learning and possibly influence academic performance in medical education [7, 34–36]. Similar to previous studies [37, 38], we found that a majority of the students in the COVID-19 year favoured asynchronous online lectures over traditional on-site lectures for many investigated aspects (Table 2). However, we found that the asynchronous online lecture alone did not lead to higher student academic performance than the traditional on-site lecture. Although asynchronous online lectures have shown many benefits, only a half of our students admitted that they preferred learning by the asynchronous online lecture to the traditional lecture for the subsequent academic year. This finding is logically incompatible with most studies, reviewed by Tang et al. [34], that indicated the students’ satisfaction towards the online teaching method. The possible explanations might include the diversity of students’ learning preference, technical issues (e.g. the instability of the e-learning platform and errors in internet connection), and the non-interactive nature of asynchronous online learning between teachers and students, as mentioned in previous studies [14, 16, 25, 38].

The strength of our study was the large number of students, i.e., 613 students enrolled in the study. However, there are two major limitations. Firstly, our study partly employed a retrospective design that limits the determination of the direct causal relationship between teaching methods and the academic achievement of students. Secondly, since all students in the same academic year were taught by the same teacher with the same teaching method in each topic (to limit the chance of inequality of knowledge distribution), we could not compare the effects of teaching methods or teachers on academic performance of students from the same class.

## Conclusions

Asynchronous online lecture alone might not be an effective teaching method in undergraduate medical education particularly when the country’s public health policy on physical distancing measures is required. Apart from teaching methods, teachers likely remain an essential key to the success in academic achievement and attitudes of undergraduate medical students. Moreover, we believe that the combination of asynchronous and synchronous online sessions together with a continuing development in teaching skills of academic faculties could be a potential solution in the ‘new normal’ medical education.

## Data Availability

Since students’ summative scores are personal data and we did not ask their consents to publicly publish the data, the datasets generated and/or analysed during the current study are not publicly available but are available from the corresponding author on reasonable request.

## Abbreviations

COVID-19: Coronavirus disease 2019
KR-20: Kuder-Richardson 20
Q&A: Question-and-answer
SBA: Single best answer
